# Over-the-Counter Hearing Aids: A Lost Decade for Change

**DOI:** 10.1155/2015/827463

**Published:** 2015-10-18

**Authors:** Zoe Yee Ting Chan, Bradley McPherson

**Affiliations:** Division of Speech and Hearing Sciences, Faculty of Education, University of Hong Kong, Pokfulam, Hong Kong

## Abstract

*Background*. Hearing aids sold directly to consumers in retail stores or through the internet, without individual prescription by audiological professionals, are termed over-the-counter (OTC) devices. This study aimed to determine whether there was any change in the electroacoustic characteristics of OTC devices compared to research carried out a decade earlier. The previous results indicated that most OTC devices were low-frequency-emphasis devices and were unsuitable for elderly people with presbycusis, who were likely to be the major consumers of these products. *Methods*. Ten OTC devices were selected and their electroacoustic performance was measured. Appropriate clients for the OTC devices were derived, using four linear prescription formulae, and OTC suitability for elderly persons with presbycusis was investigated. *Results*. OTC electroacoustic characteristics were similar to those in the earlier study. Most OTC devices were not acoustically appropriate for potential consumers with presbycusis. Although several of the devices could match prescriptive targets for individuals with presbycusis, their poor electroacoustic performance—including ineffective volume control function, high equivalent input noise, and irregular frequency response—may override their potential benefit. *Conclusion*. The low-cost OTC devices were generally not suitable for the main consumers of these products, and there has been little improvement in the appropriateness of these devices over the past decade.

## 1. Background

Hearing aids that are sold directly to consumers in retail shops or through the internet, without customized prescription by audiological professionals, are termed over-the-counter (OTC) hearing aids [1, 2]. People who purchase OTC hearing aids do not receive the potential benefits provided by professional service, which include audiological assessment, counseling, hearing aid selection, hearing aid fitting, and hearing aid orientation. Without any prior audiological assessment, unnecessary amplification or delay in diagnosis of otologic problems may result [3]. In addition, the amplification characteristics of a hearing aid may not be appropriate for the client if the hearing aid is not programmed according to the individual's hearing loss.

In many developed economies, the sale of hearing aids is regulated. For example, in the United States, the provision of hearing aids is under the regulation of the United States Food and Drug Administration. Only licensed hearing healthcare professionals can provide hearing aids. Purchasers have to show a recent medical statement proving that they are hearing aid candidates or sign a waiver stating that they declined medical evaluation of their hearing loss before receiving the hearing aids [4]. These regulations intend to protect hearing aid users from any undiagnosed ear disorders and inappropriate amplification [3]. However, in numerous jurisdictions, there is no regulation of hearing aid sales, and this is the case in Hong Kong, as in many Asian localities. In Hong Kong, OTC hearing aids can be purchased in rehabilitation aid shops, electrical appliance stores, and general department stores and through the internet.

### 1.1. Elderly People as Potential Consumers of OTC Hearing Aids

An informal survey conducted in Hong Kong by Cheng [5] indicated that customers who purchased OTC hearing aids were primarily elderly people. The main reason for purchasing OTC hearing devices is probably their low cost as they are more affordable than conventional custom hearing aids. The cost of OTC hearing devices is variable but very often less than $US250; in contrast, the cost of conventional custom hearing aids is often above $US700 in Hong Kong. In Hong Kong, the income of elderly people with low socioeconomic status mainly comes from government allowances for elderly individuals, and monthly income is about US$145 to US$282 [6]. Cost has been noted to be a major barrier to hearing aid use amongst elderly people in Hong Kong [7]. Although the Public Hospital Authority in Hong Kong provides subsidized, conventional hearing aids that often cost less than $US160, patient's first appointment waiting time is lengthy—approximately 23 to 85 weeks for a new case [8]. After the first physician visit, patients require further appointments for audiological assessment and hearing aid prescription/fitting, and this long waiting period may be another factor encouraging purchase of OTC devices.

### 1.2. Prevalence of Presbycusis and Its Common Audiometric Configuration

Presbycusis is a very common problem in the elderly population in both developed and developing countries [9]. According to the World Health Organization, the prevalence of hearing loss is approximately 33% among the global elderly population aged above 65 years [10]. In Hong Kong, it was estimated that the prevalence of presbycusis with moderate to profound hearing loss was 37.1% [11]. The common pattern of presbycusis is high frequency hearing loss and the degree of sensorineural hearing loss generally ranges from mild to moderately severe [12–15].

### 1.3. Electroacoustic Characteristics of OTC Hearing Aids in Previous Studies

Elderly people are likely to be the major users of OTC hearing aids. However, previous literature indicates that the quality of the OTC hearing aids and their effectiveness in matching the amplification needs of elderly people with presbycusis may be questionable.

#### 1.3.1. Input-Output Characteristics

Two previous studies, one undertaken in Hong Kong (Cheng and McPherson) [2] and one in North America (Callaway and Punch) [1], found that all low-cost OTC hearing aids sampled were linear hearing aids. Linear amplification has an advantage in preserving the natural loudness difference in input signals. However, it is not suitable to people with sensorineural hearing loss who have recruitment [16]. Quiet sounds may not have enough gain while loud sounds may have too much gain and it is impractical for users to adjust a volume control continuously in order to maintain an appropriate gain in an acoustically dynamic environment. In addition, Cheng and McPherson [2] found that the volume control range was limited in some OTC hearing aids, making it difficult to adjust gain to appropriate levels. The peak clipping system associated with linear hearing aids also causes distortion to high level input signals [2].

#### 1.3.2. Frequency Response

Most of the low-cost OTC hearing aids investigated in previous studies were low-frequency emphasis hearing aids with little gain or even no gain in high frequencies [1, 2]. Therefore, the OTC hearing aids tested are not suitable for elderly people with presbycusis, who typically have high-frequency, sloping hearing loss [12–15]. Speech understanding for individuals with presbycusis is not likely to be improved with low-frequency-emphasis hearing devices since consonants may remain inaudible with little gain, and amplified low-frequency background noise upwardly masks higher frequency sounds. In addition, overamplified low-frequency sounds may cause a perception of increased noise and potentially be harmful to residual hearing [1, 2]. Moreover, around half of the hearing devices tested in the two known OTC hearing aid studies showed sharp peaks of 8 dB or more in their frequency response [1, 2]. Both sound quality and speech intelligibility may be degraded with this type of response and the likelihood of feedback will also increase [16].

#### 1.3.3. Equivalent Input Noise

EIN is the internal electronic noise of the hearing aid and it becomes audible and disturbing if it is too high [17]. Some of the hearing aids examined in previous studies had very high EIN that exceeded the 28 dB SPL target maximum set by the ANSI S3.22-1987 standard [1, 2]. As a result, some OTC hearing aids can generate internal noise that is perceptible to users.

#### 1.3.4. Total Harmonic Distortion

THD reflects the amount of harmonic distortion generated by a hearing aid [16]. The THD of the OTC hearing aids tested in the two previous studies was generally within acceptable levels [1, 2].

#### 1.3.5. Acoustic Feedback

Cheng and McPherson [1, 2] reported that the sampled OTC hearing aids could generally be turned to maximum output without feedback. However, this was probably related to their poor high frequency amplification characteristics and also because the hearing devices tested at that time were mainly body worn and had a long feedback path [2].

All of the above electroacoustic characteristics of OTC hearing aids were based on low-cost models retailing for less than US$100 each. The electroacoustic performance of some higher cost OTC hearing aids may be more suitable to people with presbycusis [1, 18].

### 1.4. Aims of the Present Study

Previously studied low-cost OTC hearing aids generally were incapable of providing sufficient appropriate gain to elderly people with presbycusis and their overall electroacoustic performance was not satisfactory [1, 2]. However, these studies were conducted over 14 years ago in Hong Kong and 6 years ago in North America. The performance of OTC devices may be improved with advancing technologies and nowadays such instruments may be more suitable for elderly consumers.

The present study aimed to determine whether there was any change in the electroacoustic characteristics of OTC hearing aids available in Hong Kong over the past decade. The electroacoustic performance of the current generation of OTC hearing aids in Hong Kong was examined. In addition, the study also aimed to determine the potential client groups for the OTC hearing aids and whether recent OTC hearing aids are appropriate for elderly people with presbycusis. The results obtained may have important implications for those who plan to purchase OTC hearing aids and for audiologists and other hearing health professionals advising patients with hearing impairment.

## 2. Methods

Ten low-cost OTC hearing aids were investigated in present study. Selection was based on their wide availability to consumers in rehabilitation aid stores, electrical appliance shops and department stores in Hong Kong. The study had three aspects: (1) Measurement of the electroacoustic performance of the OTC hearing aids. Both 2-cc coupler measurement and simulated real-ear measurement were conducted to examine the performance of the OTC hearing aids; (2) Estimation of the hearing loss that could be appropriately fitted with the OTC hearing aids using four prescriptive formulae. The four prescriptive formulas used in present study were the National Acoustic Laboratories-Revised (NAL-R), prescription of gain and output (POGO), Libby one-third gain (Libby 1/3) and Desired Sensation Level 4.0 (DSL 4.0); and (3) Determination of whether the OTC hearing aids could be appropriately fit to adults with typical sensorineural hearing loss caused by presbycusis, using the NAL-R prescriptive formula. The NAL-R formula was selected because this formula is a relatively widely used method among linear prescriptive formulae [1]. It was used in both Hong Kong [2] and North American [1] research. Moreover, the NAL-R formula was designed for fitting amplification to people with mild to moderately severe hearing loss, so that it is appropriate for fitting people with typical presbycusis hearing loss configurations [19]. For comparison purposes, the methods used generally followed those of previous studies [1, 2], but an updated American National Standards Institute (ANSI) hearing aid specification standard, ANSI S3.22-2009, was used.

### 2.1. Equipment

#### 2.1.1. Over-the-Counter Hearing Aids

**Figure 1:**
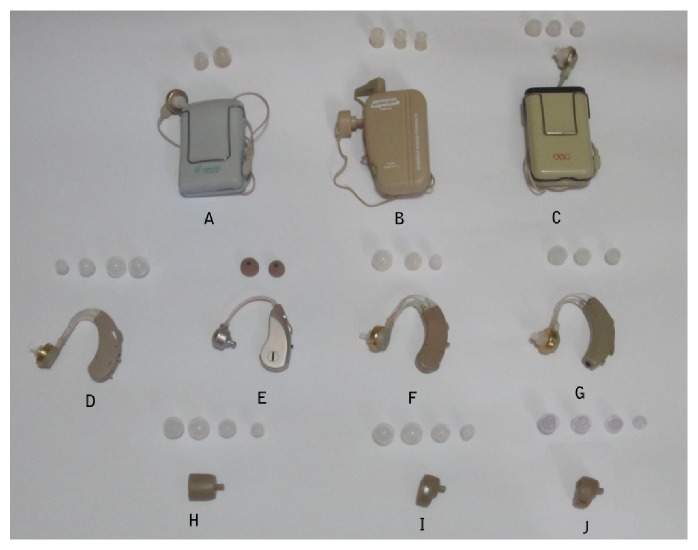
Over-the-counter hearing aids used in the present study. Top row: A: LingYin HA 611B; B: Hopewell HAP-40; C: Axwa EX-12D. Middle row: D: JNC-MHA-BTE130; E: UP-6411; F: ShengDe V-163; G: Axwa OM-188. Bottom row: H: Powertone HAP-F883; I: JNC-MHA-ITE 110; J: Axon K-80.

The ten OTC hearing aids investigated in the present study were (A) LingYin HA 611B; (B) Hopewell HAP-40; (C) Axwa EX-12D; (D) JNC-MHA-BTE130; (E) UP-6411; (F) ShengDe V-163; (G) Axwa OM-188; (H) Powertone HAP-F883; (I) JNC-MHA-ITE 110; and (J) Axon K-80. All of them were low-cost OTC hearing aids costing less than US$115 each. Moreover, they were not investigated in a previous OTC hearing aid study in Hong Kong [2]. The ten OTC hearing aids and their characteristics are shown in Figure 1 and Table 1, respectively. Three body-worn (BW), four behind-the-ear (BTE), and three in-the-ear (ITE) OTC hearing aids were included in the present study. Two of the hearing aids, Ling Yin HA 611B and Axwa EX-12D, have two and three tone controls, respectively. Therefore, there were thirteen testing conditions in the present study.

Each OTC hearing aid was provided with, on average, three stock ear domes of different sizes by the manufacturer. The outer diameter of the domes ranged from 0.7 to 1.3 cm, with a dome with outer diameter of 1.0 cm being the most commonly available size. To standardize the measurements, domes with outer diameter of 1.0 cm or the nearest available size were used in the electroacoustic measurements.

#### 2.1.2. Measurement Equipment

All of the electroacoustic measurements were conducted with a Fonix 7000 Hearing Aid Test System in a Fonix 7020 sound chamber (Frye Electronics, Tigard, OR). An HA-1 coupler was used in the 2-cc coupler measurement, as an HA-1 coupler is recommended to measure hearing aids with attached molds [1, 20]. In a simulated real-ear measurement component of the study, a Knowles Electronic Manikin for Acoustic Research (KEMAR; Knowles, Elk Grove, IL) was used to simulate the real-ear condition. KEMAR measures provide similar acoustic characteristics to measurement on a real person because the manikin can provide pinna, head, and torso effects; also it can simulate the impedance characteristics of the real ear, which changes with frequency [21]. Using KEMAR in measurements also avoids potentially loud intensity sound exposure to real listeners when an OTC hearing aid volume control is turned to a high output level or when feedback occurs.

### 2.2. Procedure

#### 2.2.1. Electroacoustic Measurements

**Figure 2:**
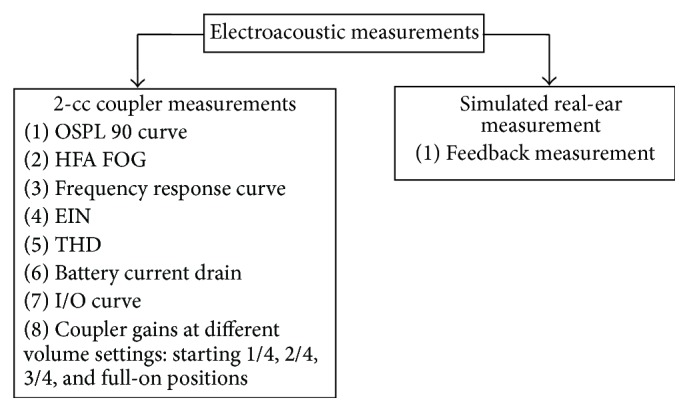
Electroacoustic measurements conducted on OTC hearing aids. OSPL 90: maximum sound pressure level output; HFA FOG; high frequency average full-on gain; EIN: equivalent input noise; THD: total harmonic distortion; I/O: input/output.

Both 2-cc coupler and simulated real-ear measurements were performed.  Figure 2 shows the full range of measures conducted.

In the 2-cc coupler measurements, the OTC hearing aids were tested according to the ANSI S3.22-2009 hearing aid specification standard [22]. Leveling of the test equipment with the equivalent substitution method was carried out before all measurements [22]. The dome portion of the hearing aid was attached to an HA-1 2-cc coupler for measuring the electroacoustic performance of hearing aid. Output sound pressure level-90 (OSPL-90) curves, high-frequency average full-on gain (HFA FOG), frequency response curves, equivalent input noise (EIN), total harmonic distortion (THD), battery current drain, and input-output curves (I/O curve) were measured with the OTC hearing aids. The battery life for each hearing aid was estimated based on the battery current drain and the capacity of battery used [1].

In addition, the output sound pressure level and the gain at different volume settings were measured. The volume control wheels of the hearing aids were divided into 4 equal portions and the gains were measured at the starting, 1/4, 1/2, 3/4, and the full-on positions [2]. Composite noise of 50 dB SPL was used as test signal to avoid saturation of the OTC hearing aids [2].

In the simulated real-ear feedback measurements, the hearing aids were tested on KEMAR's right pinna. The loudspeaker was located at an azimuth angle of 45° and 30 cm from KEMAR [20]. The center of the loudspeaker was at the same level as the midpoint of the KEMAR pinna. To simulate a normal conversational situation, the input signal used was digital speech at 60 dB SPL [2]. The volume control was rotated until feedback was detected by the normal hearing first author who stood at 25 cm behind KEMAR or when abnormal peaks began to appear in the frequency response during testing [2]. The volume settings that the OTC hearing aids could achieve before audible or visible feedback occurred were measured.

#### 2.2.2. Appropriate Hearing Loss for OTC Hearing Aids

The hypothetical hearing losses that could be appropriately prescribed with the OTC hearing aids were estimated. These estimates were based on the 2-cc coupler gain at the full-on position for each device and were derived using four prescription formulae [2]. The reserve gains recommended by the four selected fitting formulae were allowed for [19].

#### 2.2.3. Fitting OTC Hearing Aids for Presbycusis Using NAL-R Formula

The average hearing thresholds of elderly people were estimated based on the study by Stenklev and Laukli [23], who surveyed the hearing levels of elderly people aged 60 or above in Norway. The ratio of male to female participants was 1.1 : 1, and average hearing thresholds were estimated by averaging the mean pure-tone hearing thresholds of both ears and both genders. The estimated hearing thresholds are showed in Table 2. These data were chosen because (1) the sample size was reasonably large, having included 232 subjects; (2) the data were relatively up-to-date when compared with the data used in previous work [2]; and (3) the data were collected in a sound-attenuating room meeting international standards.

Target 2-cc coupler full-on gains were generated by the NAL-R prescription formula based on the estimated average hearing thresholds of an elderly person shown in Table 2. The calculated target 2-cc coupler full-on gains were compared with the measured 2-cc coupler full-on gains for the OTC hearing aids to determine whether the amplification characteristics of the OTC hearing aids could appropriately fit people with presbycusis [1]. In the present study, the tolerances for matching the prescriptive targets were set to ±5 dB at 250 Hz, 500 Hz, 1 kHz, and 2 kHz and ±8 dB at 3 kHz and 4 kHz [24]. If the OTC hearing aid matched the prescriptive target for four frequencies or more, that hearing aid was judged to satisfactorily meet the amplification needs of elderly people with a typical hearing loss associated with presbycusis [1]. If the OTC hearing aid failed to match the prescription target under the above criterion, a higher tolerance of ±10 dB at all frequencies was used to determine whether they could meet this less strict criterion [25].

## 3. Results

### 3.1. Electroacoustic Measurements

#### 3.1.1. 2-cc Coupler Measurements

2-cc coupler measurement results for the ten OTC hearing aids are summarized in Table 3. The maximum OSPL 90 was 125 to 130 dB SPL for most of the OTC hearing aids, except for OTC E, I, and J which had their maximum OSPL 90 at approximately 115 dB SPL. Most of the OTC hearing aids showed their peak response at around 1400 Hz to 2000 Hz, while OTC B, H, and J had their maximum responses at 700 Hz to 800 Hz.

**Figure 3:**
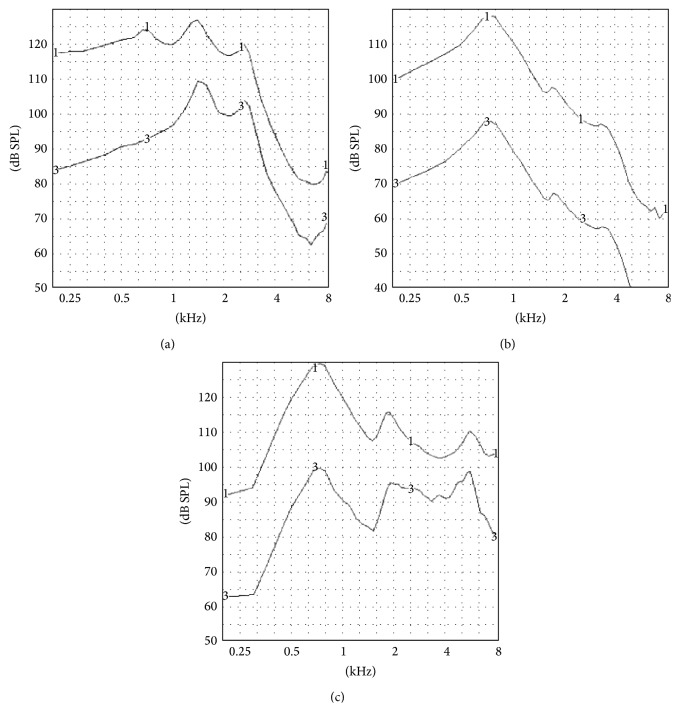
OSPL 90 curves (1) and frequency response curves (3) of OTC hearing aids: (a) C tone N; (b) J; (c) B.

All OTC hearing aid frequency response curves showed high frequency limits up to 4000 Hz, except for OTC C (all tones), D, and F. The shape of the frequency response curve for OTC B differed from that usually found in hearing aids. The OSPL 90 curves and frequency response curves of three OTC hearing aids (OTC C tone N, OTC J, and OTC B) are shown in Figure 3 and represent the group of OTC hearing aids with a peak response at mid frequencies, the group with a peak response at low frequencies, and the device with an irregular frequency response, respectively.

**Figure 4:**
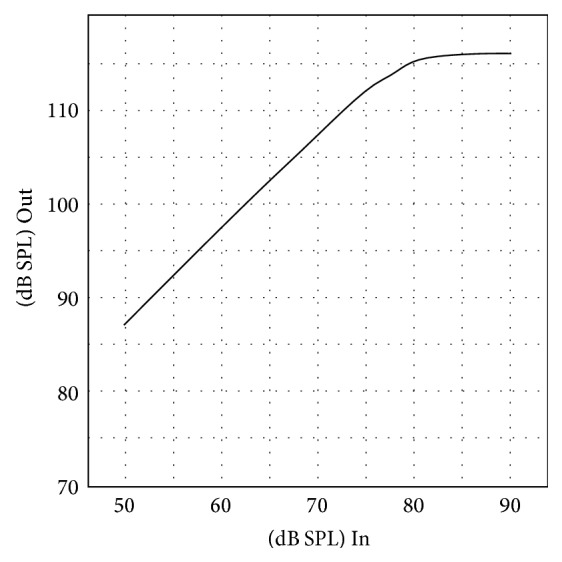
I/O curve of OTC hearing aid G.

**Figure 5:**
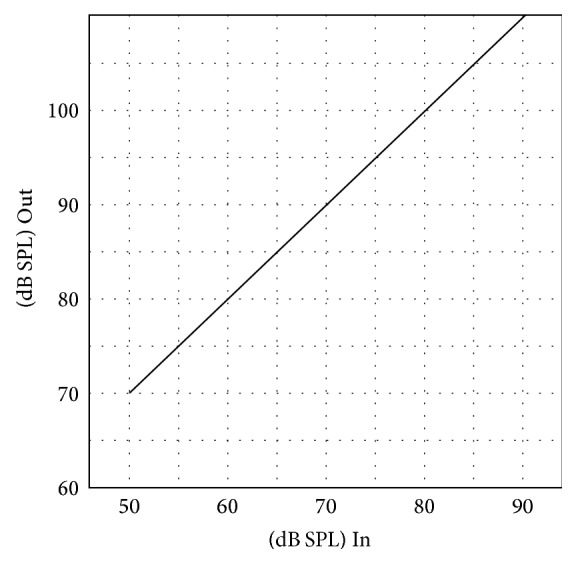
I/O curve of OTC hearing aid E.

All of the OTC hearing aids investigated were linear hearing aids and most of them showed peak clipping at high input levels.  Figure 4 displays the I/O curve of OTC G, which shows this typical peak clipping effect. The output level of the OTC devices was generally limited to around 110 dB to 120 dB SPL. However, some of the low-gain hearing aids were not saturated even at a 90 dB input level, such as OTC E, H, I, and J.  Figure 5 shows the I/O curve of OTC E and it can be noted that no saturation occurred.

Volume control characteristics are shown in Table 4. The volume range between different volume settings was measured and the percentage of total gain at different volume settings was calculated.

#### 3.1.2. Simulated Real-Ear Feedback Measurement

In the feedback measurement, none of the OTC hearing aids exhibited feedback problems even at a full-on volume position.

#### 3.1.3. Statistical Analysis

The peak frequency and peak sound pressure level at OSPL 90, the HFA FOG, and EIN in the present study were compared with those parameters in Cheng and McPherson's study [2] using an independent *t*-test analysis. In these four parameters, the input signals used in measuring HFA FOG and EIN are different between ANSI 3.22-1987 and ANSI 3.22-2009 standards. Nevertheless, the gain measured with either 60 dB in ANSI 3.22-1987 or 50 dB in ANSI 3.22-2009 is the same for linear hearing aids. Therefore, comparison can be made between the two studies. The results are presented in Table 5 and reveal no significant difference (*p* > 0.05) in these parameters between the present study and the previous Hong Kong OTC hearing aid study.

### 3.2. Appropriate Hearing Loss for OTC Hearing Aids

**Figure 6:**
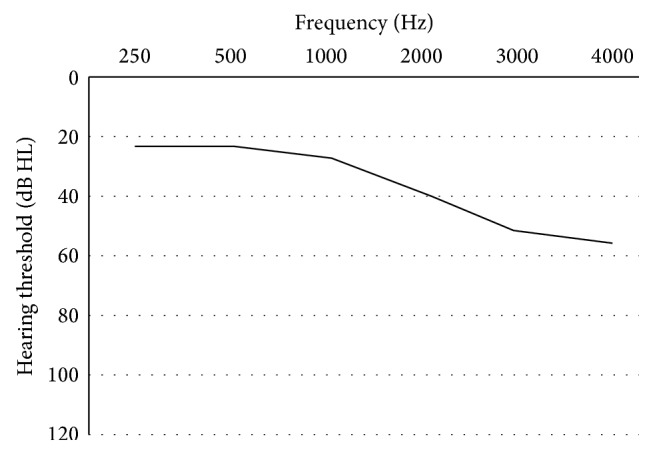
Estimated mean audiogram for presbycusis based on Stenklev and Laukli's data [23].

**Figure 7:**
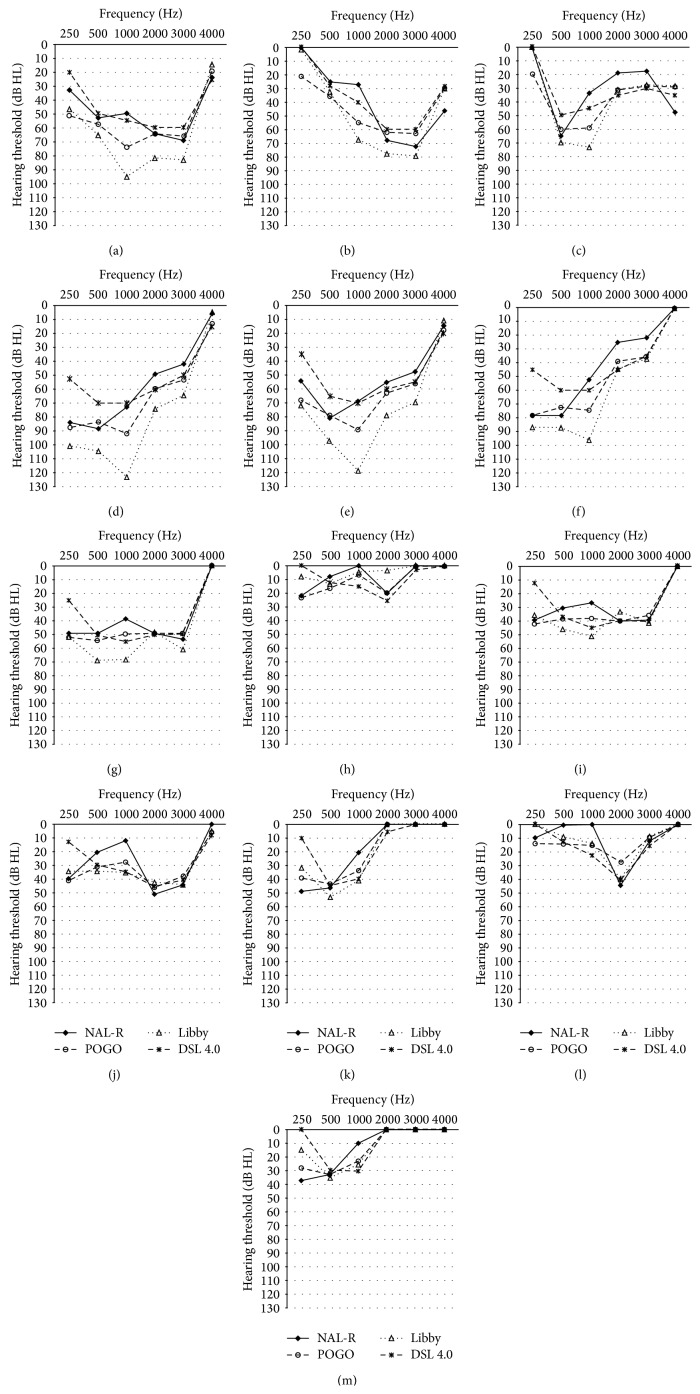
(a) Appropriate hearing loss for OTC A (tone N). (b) Appropriate hearing loss for OTC A (tone L). (c) Appropriate hearing loss for OTC B. (d) Appropriate hearing loss for OTC C (tone N). (e) Appropriate hearing loss for OTC C (tone H). (f) Appropriate hearing loss for OTC C (tone L). (g) Appropriate hearing loss for OTC D. (h) Appropriate hearing loss for OTC E. (i) Appropriate hearing loss for OTC F. (j) Appropriate hearing loss for OTC G. (k) Appropriate hearing loss for OTC H. (l) Appropriate hearing loss for OTC I. (m) Appropriate hearing loss for OTC J.

The hypothetical hearing losses estimated by the four prescription formulae when using the 2-cc coupler full-on gain data were plotted for each OTC hearing aid and are shown in Figure 7.  Figure 6, showing the mean Stenklev and Laukli audiogram for presbycusis [23], is displayed for comparison purposes. Any negative values for derived hearing thresholds were assumed to equate to normal hearing with 0 dB HL threshold [2]. The types of hearing loss that could be appropriately fit with the OTC hearing aids were generally divided into four categories: (1) sloping hearing loss up to 3000 Hz (OTC A tones N and L); (2) reverse sloping hearing loss (OTC B, C all tones, H, and J); (3) flat loss up to 3000 Hz (OTC D, F, and G); and (4) normal or minimal hearing loss (OTC E and I).

### 3.3. Fitting OTC Hearing Aids for Presbycusis Using NAL-R Formula

Table 6 summarizes findings for the stricter prescription matching criterion and Table 7 for the looser criterion. OTC A (tone L), F, and G were the only hearing aids that could meet the amplification needs of elderly people with presbycusis if the stricter criterion was used. If the looser criterion for matching targets was used, OTC B also matched the amplification needs for presbycusis.

## 4. Discussion

### 4.1. Electroacoustic Measurement Findings

#### 4.1.1. OSPL 90 Curves and Frequency Response Curves

Most of the OTC hearing aids showed their peak response at the mid frequencies, while OTC B, H, and J had their maximum response at the low frequencies. Excessive amplification in the low frequencies will increase the adverse effects of background noise and additionally increase the possibility of upward spread of masking by low-frequency speech components [21]. Hence, speech intelligibility will be reduced with this pattern of amplification.

Despite most OTC hearing aids having a frequency range up to 4000 Hz, there was little or no usable gain at or above 4000 Hz. Some frequency responses dropped abruptly at about 4000 Hz, such as OTC A (tone N and tone L), OTC E, and OTC I. In the present study, all the BTE and ITE hearing aids examined could not provide adequate high frequency amplification. BW was the only style that could provide 15 dB or more gain at 4000 Hz.

Narrow peaks with approximately 10 dB amplitude were observed in the high frequency region of the frequency responses for OTC A (tone N and tone L), OTC D, OTC E, and OTC F. According to Dillon and Macrae [26] and van Buuren et al. [27], narrow peaks with 6 dB amplitude or more in the frequency response evoke a negative response from hearing aid users. Although OTC B did not show narrow peaks in the frequency response, its frequency response was quite irregular with several broad peaks. The smoothness of a hearing aid frequency response has been found to have a positive relationship with speech intelligibility and sound quality [27–29].

#### 4.1.2. High-Frequency Average Full-on Gain (HFA FOG)

The HFA FOG was generally higher in the BW styles and lower in the ITE styles. The mean HFA FOG was 43.3 dB in BW, 29.9 dB in BTE, and 14.2 dB in ITE hearing aids. HFA FOG is the average FOG at 1000 Hz, 1600 Hz, and 2500 Hz and is used in the ANSI standard because these frequencies are very important for speech intelligibility and because most hearing aids generate significant amount of gain at these frequencies [22]. However, more than half of the OTC hearing aids exhibited a characteristic of “special-purpose hearing aids” in that their peak FOGs at any frequency were 15 dB higher than the FOG at any of the HFA frequencies [22]. For example, OTC J had a very low HFA FOG of 7.6 dB, but its peak FOG at 700 Hz was 29.3 dB. Thus, OTC J was not a low-gain hearing aid, but it amplified particular low frequencies rather than the typical HFA frequencies. Some of the OTC hearing aids may be designed for specific purposes, but these special purposes were not stated in the packaging or user manuals. This is similar to the results for OTC hearing aids studied by Callaway and Punch [1], who found that all of the low-cost OTC hearing aids they tested could be classified as special-purpose hearing aids as defined by ANSI S3.22-2009.

#### 4.1.3. Input-Output Characteristics

All of the OTC hearing aids investigated were linear hearing aids and most of them showed peak clipping at high input levels. As mentioned before, linear hearing aids are often not suitable for people with sensorineural hearing loss [16]. Moreover, a peak clipping system will introduce more distortion when compared with a compression limiting system [30]. Peak clipping will degrade both the sound quality and speech intelligibility of loud inputs to a greater extent than a compression limiting system [30, 31].

#### 4.1.4. Equivalent Input Noise

In the present results, the EIN ranged from 24.9 to 52.9 dB SPL, with a mean value of 33.1 dB SPL. The extremely high EINs found in OTC J and OTC H were probably measurement artifacts due to their low HAF gain [16]. According to ANSI S3.22-1987 standards [1], EIN should be limited at 28 dB SPL or less. Only three of the hearing aid measurement conditions were within the EIN limit set by ANSI 3.22-1987, which were OTC C (tones N and H) and OTC F. As a result, some OTC hearing aids will generate internal noise that is perceptible to users and may be high enough to elicit user rejection [32].

#### 4.1.5. Total Harmonic Distortion

THD values below 5% and not more than 10% were recommended by Dillon and Macrae [26]. Most of the OTC hearing aids examined therefore had acceptable THD levels, except for OTC I. The manufacturer of OTC I specified the THD level to be 10% or lower without stating the tested frequencies. However, the THD levels at 500 Hz, 800 Hz, and 1600 Hz were all higher than 10% and THD level was 46.5% at 800 Hz. THD levels should not exceed manufacturer specification by more than 3%; otherwise, the hearing aid may have malfunctioned [22]. Moreover, another hearing aid (OTC G) had an intermittent response and its volume control was hard to rotate. Quality control is a potential problem for OTC hearing aid purchasers since salespeople may not have sufficient knowledge to check hearing aid function prior to sale.

#### 4.1.6. Battery Life

The estimated battery life of all the OTC hearing aids was acceptable, ranging from 142 hours to 307 hours. This was probably because they used a relatively larger size of battery than conventional hearing aids. All BTE hearing aids investigated in the present study used a 675 zinc air battery, which is rarely used in modern, conventional BTE hearing aids except for high power BTE hearing aids. Therefore, the BTE style OTC hearing aids were larger in size when compared to modern conventional BTE hearing aids of similar gain.

#### 4.1.7. Volume Control Characteristics

**Figure 8:**
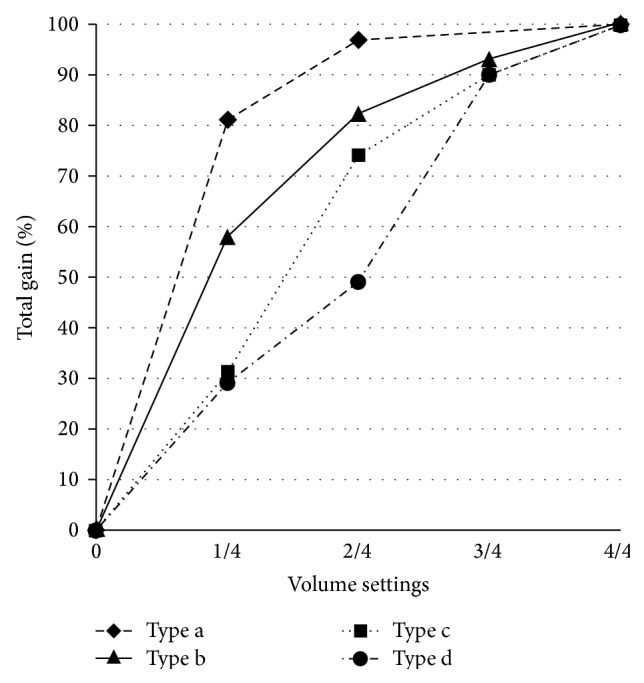
OTC hearing aid volume control gain characteristics.

The mean volume range of the OTC hearing aids was 31.3 dB, indicating that there was about 31 dB flexibility in volume adjustment. However, the volume control (VC) was often not very effective as gain did not increase proportionally with volume control wheel adjustment. Half of the OTC hearing aids attained more than 50% of total gain at 1/4 volume setting. The VC characteristics could be divided into four types: (a) OTC A and F attained at least 75% of total gain at 1/4 volume setting; (b) OTC D, G, and J attained approximately 50% of total gain at 1/4 volume setting and the gain increased rapidly to at least 80% of total gain at 2/4 volume setting; (c) OTC E, H, and I attained less than 50% of total gain at 1/4 volume settings, but the gain increased rapidly to at least 70% of total gain at 2/4 volume setting; (d) OTC B and C attained approximately 50% of total gain at 2/4 volume setting. All OTC hearing aids reached nearly maximum gain at 3/4 volume settings. The percentages of total gain at different volume settings were plotted in Figure 8 for the four types of VC; one representative was chosen from each VC type.

Type d was the most effective VC and type a was the least effective among the four types of VC. OTC A had the poorest VC among all OTC hearing aids. Although OTC A (tone N) had a volume range of 30 dB between the 0/4 to 4/4 volume settings, there was only 6 dB difference between 1/4 and 4/4 volume settings. Consequently, it could be difficult for a hearing aid user to adjust the volume to an optimum listening level as rotation of the volume control will result in either too much or too little change in gain.

In addition, hearing aids are not typically designed to operate at the full-on position in usual situations. Reserve gain should be allowed for hearing aid users, so that they have the freedom to adjust for different listening environments. Most prescription formulae suggest a reserve gain of 10 to 15 dB [19]. It would be optimal if the hearing aid can provide appropriate gain when it is operated at the mid VC setting. However, only OTC B and C were able to provide a reserve gain of 10 to 15 dB at the mid VC setting.

#### 4.1.8. Acoustic Feedback

In the simulated real-ear measurement, all of the OTC hearing aids could turn to full-on gain without feedback occurring. This was probably because the BTE hearing aids and ITE hearing aids were low-gain hearing aids with little output at high frequencies. Moreover, the long feedback pathway in the BW style may reduce the likelihood of feedback [2].

#### 4.1.9. Comparison of the OTC Hearing Aids in the Present Study with Those in Previous Studies

In the present study, the performances of the OTC hearing aids were generally similar to those investigated by Cheng and McPherson [2] and the low-cost OTC hearing aids investigated by Callaway and Punch [1]. All were linear hearing aids and most of them had little usable high frequency gain. Problematic peaks were observed in the high frequency region of the frequency response curve in some of the hearing aids. EIN was still a concern for many OTC hearing aids. THD and battery life were generally acceptable in the present study and results were comparable with previous studies. Statistical analysis on the peak frequency and peak sound pressure level of OSPL 90, the HFA FOG, and EIN revealed no significant differences between the OTC hearing aids in the present study and those in the Cheng and McPherson [2] study.

### 4.2. Appropriate Hearing Loss for OTC Hearing Aids

The appropriate hearing loss for each OTC hearing aid was estimated using the four prescription formulae and illustrated in Figure 7. Only OTC A (tone L) and OTC B were able to provide enough amplification for a mild hearing loss at 4000 Hz; all other OTC hearing aids were only suitable for people with normal hearing thresholds at 4000 Hz. In the present study, approximately half of the hearing aid test conditions (OTC B, OTC C tone N, tone H, and tone L, OTC H, and OTC J) were suitable for people with reverse sloping hearing loss. Similarly, the majority of the OTC hearing aids investigated by Cheng and McPherson [2] revealed this phenomenon. Individuals with Meniere's disease or early stage otosclerosis may be potential clients for these OTC hearing aids [33]. The hearing loss estimated for OTC A showed a sloping configuration, especially when operated at tone L, and thus OTC A was the most appropriate device to fit presbycusis. The hypothetical appropriate hearing loss estimated from the gain of the three BTE hearing aids (OTC D, F, and G) revealed a flat hearing loss up to 3000 Hz. Therefore, these BTE hearing aids could improve the loudness of speech for people with mild to moderate flat hearing loss, but they might not provide sufficient improvement in speech intelligibility since they did not provide adequate gain at 4000 Hz. OTC I was suitable for people with mild to moderate hearing loss at 2000 Hz, but this type of hearing loss is comparatively rare. OTC E was suitable for people with relatively normal hearing sensitivity only due to its low gain.

Although the variety of appropriate hearing losses for OTC hearing aids was greater in present study than in that by Cheng and McPherson [2], it may be the result of random product selection in the two studies. Some of the OTC hearing aids in the present study were suitable for people with sloping or flat hearing loss. Nevertheless, reverse sloping hearing loss was still the most common audiological configuration appropriate for the OTC hearing aids. However, the major users of OTC hearing aids are elderly people who have high frequency sloping hearing loss [14, 18]. These individuals typically experience hearing difficulties in noisy environments since they have reduced frequency resolution ability [21]. If the elderly person with presbycusis wears a low-frequency emphasis OTC hearing aid, their speech understanding in noise will further deteriorate due to upward spread of masking caused by amplified background noise and amplified low-frequency speech components [2].

There was a limitation in the estimation of hypothetical hearing loss in the present study since the prescription formulae included reserve gain of 10 to 15 dB and assumed the hearing aids were operated at mid volume settings [21]. However, most of the OTC hearing aids did not have enough reserve gain when operated at the mid volume settings. Therefore, Figure 7 reflects the hearing loss that can be fit appropriately with the hearing aid when the hearing aid was operated at the volume setting which is 10 to 15 dB lower than the full-on gain. Some of the devices were therefore operating at 0/4 to 1/4 volume settings.

### 4.3. Fitting OTC Hearing Aids for Presbycusis Using NAL-R Formula

OTC A (tone L), F, and G were the only hearing aids that could meet the amplification needs of elderly people with presbycusis if a strict fitting outcome criterion was used. If a looser criterion of matching targets was used, OTC B also could be considered to match the amplification needs for presbycusis. Generally, BW styles could match the prescriptive targets at the high frequencies, but they provided too much low-frequency gain at the same time; thus they usually failed to match the targets at low frequencies; in contrast, BTE and ITE styles could match the fitting targets at low frequencies, but they usually failed to provide sufficient amplification in the high frequencies.

Although OTC A (tone L), F, and G could fit a hypothetical presbycusis using the NAL-R formula, they provided 15 dB reserve gain only when they were operated at 0/4 to 1/4 VC settings. These three hearing aids had type a and type b VC characteristics, such that the gains increase abruptly between 0/4 and 1/4 VC settings. This attribute would make it difficult for wearers to adjust the gain to an appropriate level, especially for elderly people with poor manual dexterity. Overamplification or underamplification would be the likely result due to the poor VC range in these hearing aids [2]. OTC B had relatively more effective VC, but its frequency response curve was quite irregular which may affect sound quality and speech intelligibility. Moreover, OTC B failed to meet the prescriptive target at 500 Hz due to overamplification at that frequency. In summary, some of the OTC hearing aids could match the prescriptive targets of presbycusis, but, on the other hand, their poor VC performance and inappropriate frequency response made them unsuitable for elderly people with typical presbycusis audiometric configuration. Poor benefit, increased background noise, poor sound quality, and the need to adjust the volume control are some of the major reasons that hearing aid users do not use their hearing aids [34]. Negative experience with an OTC hearing aid that has these characteristics may keep wearers from further amplification device purchase and users may label all “hearing aids” as useless. Consumers also require information on how to successfully wear and adapt to a hearing instrument. In the present study, a Chinese operation manual version was not available with half of the OTC hearing aids, making it difficult for many elderly people in Hong Kong to learn correct fitting procedures.

## 5. Conclusion

The present findings indicate that the electroacoustic characteristics of the selected, current generation OTC hearing aids are similar to those in previous studies over the past decades. There is no major improvement shown in the performance of OTC hearing aids over the years. All were linear hearing aids with less than optimal volume controls. Most of them showed unacceptable electroacoustic performance, such as sharp peaks in the high frequency region of frequency response, low HFA gain, poor amplification in high frequencies, and/or high EIN. However, THD levels, battery life, and feedback were generally not problematic in the OTC hearing aids.

Reverse sloping hearing loss was still the most prevalent type of hearing loss that could be appropriately fit with the OTC hearing aids. This type of low-frequency emphasis hearing aid is not suitable for most people with presbycusis, who typically show high frequency sloping hearing loss. The prescriptive targets for presbycusis were generated using the NAL-R formula. Four of the hearing aid test conditions could match the prescriptive targets at four or more frequencies, and they were judged to meet the amplification needs of elderly people with presbycusis. However, their electroacoustic performance, such as ineffective volume control, high EIN, and irregular frequency response, may override their benefit in matching the amplification needs of clients with presbycusis. For example, the appropriate hearing loss derived from OTC A was a sloping hearing loss; thus OTC A was potentially suitable for elderly individuals with hearing loss associated with presbycusis. Nevertheless, OTC A had inadequate volume control parameters and attained approximately 80% of total gain at 1/4 volume setting, making it difficult for elderly wearers to adjust gain to an appropriate level.

In summary, the low-cost OTC hearing aids investigated in the present study were not considered suitable for elderly people with presbycusis, who are likely to be the major users of OTC hearing aids. The inadequate performance of such OTC hearing aids may cause wearers to decline to adopt hearing aid use. Manufacturers should consider ways to improve VC effectiveness, lower EIN, and smooth the frequency response of OTC hearing aids. Moreover, they may consider increasing the gain at high frequencies and reducing the gain at low frequencies, in line with prescription formulae guidelines. Future OTC hearing aids then may be more suitable for their major client group—elderly people with presbycusis. On the other hand, most conventional hearing aids have advanced technology, such as directional microphones, noise-reduction algorithms, and automatic volume controls, which can improve the listening experience of wearers. In addition, the hearing aid prescribed by an audiological professional can appropriately fit the user because the electroacoustic parameters are specifically adjusted according to their individual hearing loss. Although conventional hearing aids are more expensive than OTC hearing aids, the benefits brought by conventional hearing aids may far outweigh their cost. To clarify this point further research should be done, using subjective rating procedures, on the fitting experience of current OTC hearing aid users [18]. Qualitative opinions are important because objective measurements cannot fully reflect the actual performance of a device on users [35].

## Figures and Tables

**Table 1:** Summary of the characteristics of the ten OTC hearing aids.

	Models	Style	Cost ($US)	Country of manufacturer	Volume range	Special features	Operation manual	Technical specification	Battery
A	LingYin HA 611B	BW	41	China	1–5	2 tone controls (N; H)	Yes (Chinese)	Yes	AAA
B	Hopewell HAP-40	BW	22	Unknown	No marking	—	Yes (English and Spanish)	No	AAA
C	Axwa EX-12D	BW	49	China	1–8	3 tone controls (N; H; L)	Yes (English)	Yes	AA
D	JNC-MHA-BTE130	BTE	47	Korea	1–3	—	Yes (Chinese and English)	Yes	675
E	UP-6411	BTE	52	Japan^#^	1–6	—	Yes (English)	Yes	675
F	ShengDe V-163	BTE	51	China	1–4	—	Yes (Chinese)	Yes	675
G	Axwa OM-188	BTE	55	China	1–4	—	Yes (English)	Yes	675
H	Powertone HAP-F883	ITE	114	Unknown	1–5	—	Yes (English)	Yes	13
I	JNC-MHA-ITE 110	ITE	47	Korea	No marking	—	Yes (Chinese and English)	Yes	312
J	Axon K-80	ITE	37	Unknown	No marking	—	Yes (English)	Yes	312

*Note.* #: no information of manufacturer is printed on packaging, but the salesperson claimed that it was a Japanese brand; BW: body-worn; BTE: behind-the-ear; ITE: in-the-ear; N: normal; H: high; L: low; technical specification: manufacturer's information on electroacoustic characteristics of the hearing aid.

**Table 2:** Estimated hearing thresholds of elderly people based on Stenklev and Laukli data [23].

	Frequency (Hz)					
	250	500	1000	2000	3000	4000
Estimated hearing threshold (dB HL)	23.4	23.6	27.1	38.1	51.8	55.8

*Note.* These values show mean hearing thresholds of elderly people aged 60 or above, including both male and female and left and right ears.

**Table 3:** Summary of the results of OTC hearing aids: 2-cc coupler measurements.

OTC	OSPL 90		HFA FOG (dB)	Frequency range (Hz)	EIN (dB)	THD (%)			Battery life (hours)
	Peak frequency (Hz)	Peak SPL (dB SPL)				500 Hz	800 Hz	1.6 kHz	
A tone N	1600	127.6	44.0	375–4000	28.4	1.9	N/A	0.4	DNT
A tone L	1600	128.5	39.7	667–4667	28.5	N/A	N/A	0.7	DNT
B	700	129.8	29.0	354–>8000	35.2	0.3	0.1	0.7	DNT
C tone N	1400	126.6	52.8	<200–3667	26.4	2.3	N/A	0.1	DNT
C tone H	1400	126.3	52.5	<200–3667	25.5	2.4	N/A	0.3	DNT
C tone L	1400	126.1	41.8	396–3667	29.7	3.3	3.1	0.1	DNT
D	1400	129.3	37.2	<200–3667	30.6	2.1	1.2	0.5	142
E	1700	118.8	19.1	<200–4667	38.1	4.2	N/A	0.1	182
F	1400	125.9	32.6	<200–3833	24.9	1.4	N/A	0.3	233
G	1600	126.8	30.6	<200–5333	33.2	2.7	N/A	0.2	235
H	800	124.4	14.2	<200–4333	45.6	6.6	1.2	10.1	307
I	2000	113.1	20.9	<200–5000	31.4	23.5	46.5	10.8	154
J	700	118.4	7.6	<200–4667	52.9	4.8	0.8	4.6	212

*Note.* Peak SPL: peak sound pressure level; N/A: not applicable. According to the 12 dB rule, THD does not need to be measured at that frequency when its second harmonic was amplified 12 dB more than the first harmonic in the frequency response curve (Frye, 2010 [20]).

DNT: did not test. Measurement of battery current drain was not conducted since no battery substitution pills for AA and AAA battery size were available.

**Table 4:** OTC hearing aids: volume range and percentage of total gain at different volume control settings.

OTC	Volume range between different volume settings (dB SPL)				Percentage of total gain at different volume settings (%)			
	Starting to full-on	1/4 to full-on	2/4 to full-on	3/4 to full-on	1/4	2/4	3/4	Full-on
A tone N	30	6	1	0	81	97	100	100
A tone L	27	6	1	0	78	97	100	100
B	36	25	18	3	29	49	90	100
C tone N	36	20	15	2	44	59	94	100
C tone H	36	22	15	3	40	57	93	100
C tone L	35	20	14	3	44	61	92	100
D	40	19	7	2	53	82	95	100
E	19	12	4	2	40	81	90	100
F	36	9	5	2	75	87	95	100
G	33	14	6	1	58	82	93	100
H	30	18	5	1	41	83	95	100
I	22	15	6	2	31	74	90	100
J	27	12	5	2	54	80	94	100

**Table 5:** Statistical analysis of the OSPL90, HFA FOG, and EIN data between present study and Cheng and McPherson [2] study.

Parameter	Study	Number of testing conditions	Means	*p* value
OSPL 90-peak frequency	Present study	13	1361.5 Hz	0.357
	Cheng and McPherson	16	1193.8 Hz	

OSPL 90-peak sound pressure level	Present study	13	124.7 dB SPL	0.653
	Cheng and McPherson	16	125.9 dB SPL	

HFA FOG	Present study	13	32.5 dB	0.422
	Cheng and McPherson	16	36.1 dB	

EIN	Present study	13	33.1 dB SPL	0.124
	Cheng and McPherson	16	28.4 dB SPL	

**Table 6:** Judgment of matching prescriptive targets for presbycusis with stricter criterion.

OTC	Matching the prescriptive targets for presbycusis?						Match the targets at four or more frequencies?
	0.25 Hz	0.5 Hz	1 kHz	2 kHz	3 kHz	4 kHz	
A tone N	*✗*	*✗*	*✗*	*✗*	*✗*	✓	*✗*
A tone L	*✗*	✓	✓	*✗*	✓	✓	✓
B	*✗*	*✗*	✓	✓	*✗*	✓	*✗*
C tone N	*✗*	*✗*	*✗*	*✗*	✓	*✗*	*✗*
C tone H	*✗*	*✗*	*✗*	*✗*	✓	✓	*✗*
C tone L	*✗*	*✗*	*✗*	✓	✓	*✗*	*✗*
D	*✗*	*✗*	*✗*	*✗*	✓	*✗*	*✗*
E	✓	*✗*	*✗*	*✗*	*✗*	*✗*	*✗*
F	*✗*	✓	✓	✓	✓	*✗*	✓
G	✓	✓	✓	✓	✓	*✗*	✓
H	*✗*	✓	✓	*✗*	*✗*	*✗*	*✗*
I	*✗*	*✗*	*✗*	✓	*✗*	*✗*	*✗*
J	✓	✓	*✗*	*✗*	*✗*	*✗*	*✗*

*Note.* ✓: OTC hearing aid matched prescriptive target.

**Table 7:** Judgment of matching prescriptive targets for presbycusis with looser criterion.

OTC	Matching the prescriptive targets for presbycusis?						Match the targets at four or more frequencies?
	0.25 Hz	0.5 Hz	1 kHz	2 kHz	3 kHz	4 kHz	
A tone N	✓	*✗*	*✗*	*✗*	✓	✓	*✗*
A tone L	✓	✓	✓	*✗*	✓	✓	✓
B	✓	*✗*	✓	✓	✓	✓	✓
C tone N	*✗*	*✗*	*✗*	✓	✓	✓	*✗*
C tone H	*✗*	*✗*	*✗*	*✗*	✓	✓	*✗*
C tone L	*✗*	*✗*	*✗*	✓	✓	*✗*	*✗*
D	*✗*	*✗*	✓	✓	✓	*✗*	*✗*
E	✓	✓	*✗*	✓	*✗*	*✗*	*✗*
F	✓	✓	✓	✓	✓	*✗*	✓
G	✓	✓	✓	✓	✓	*✗*	✓
H	✓	✓	✓	*✗*	*✗*	*✗*	*✗*
I	✓	✓	*✗*	✓	*✗*	*✗*	*✗*
J	✓	✓	✓	*✗*	*✗*	*✗*	*✗*

*Note.* ✓: OTC hearing aid matched the prescriptive target.

## Abbreviations

OTC: Over-the-counter
EIN: Equivalent input noise
THD: Total harmonic distortion
NAL-R: National Acoustic Laboratories-Revised
POGO: Prescription of gain and output
DSL 4.0: Desired Sensation Level version 4.0
ANSI: American National Standards Institute
BW: Body-worn
BTE: Behind-the-ear
ITE: In-the-ear
KEMAR: Knowles Electronic Manikin for Acoustic Research
OSPL-90: Output sound pressure level-90 dB input
HFA FOG: High frequency average full-on gain
I/O: Input/output
VC: Volume control.
